# Antibiofilm effects of bacteriocin PCM7-4 on *Listeria monocytogenes*

**DOI:** 10.1371/journal.pone.0325109

**Published:** 2025-06-05

**Authors:** Haotian Ma, Jinju Peng, Yang Li, Ruixue Pan, Yuner Long, Yining Zhao, Yuexia Ding, Yi Ma

**Affiliations:** College of Coastal Agricultural Sciences, Guangdong Ocean University, Zhanjiang, China; Agricultural University of Athens: Geoponiko Panepistemio Athenon, GREECE

## Abstract

*Listeria monocytogenes* is a significant zoonotic pathogen capable of forming biofilms on food and other materials, representing a considerable risk to human health and animal husbandry. The use of bacteriocins as potential new antibacterial and antibiofilm reagents has attracted considerable interest. This study aimed to determine the inhibitory effects of bacteriocin PCM7−4 on *L. monocytogenes* biofilm formation. In this study, bacteriocin PCM7−4 of SICs (1/16 × MIC, 1/8 × MIC) significantly inhibited the formation of *L. monocytogenes* biofilm. Bacteriocin PCM7−4 of SICs significantly reduced the production of bacterial extracellular polysaccharides, and could decrease the bacterial motility, meanwhile, PCM7−4 significantly reduced the number and viability of bacteria within the biofilm. RT-qPCR results showed that bacteriocin PCM7−4 significantly reduced the expression of flagella, community sensing and virulence factor genes associated with biofilm formation. The results demonstrated the considerable potential of bacteriocin PCM7−4 as a therapeutic agent for the prevention and treatment of *L. monocytogenes* biofilms.

## 1. Introduction

*Listeria monocytogenes* is a Gram-positive bacterium that is widely found in agricultural, aquacultural, and food processing environments. It is an important foodborne pathogen and is regarded as one of the three most harmful zoonotic pathogens, in conjunction with *Campylobacter* and *Salmonella* and a major agent of listeriosis in animals and humans [1–3]. The risk is particularly elevated in groups of pregnant women, elderly individuals, infants and patients with immunosuppression [4]. Infection with *L. monocytogenes* has been associated with a range of serious and potentially life-threatening conditions, including diarrhoea, septicaemia, meningitis, endocarditis and encephalitis [5–7]. These risks pose a significant threat to human health and the development of livestock.

Biofilms are defined as complex associations of microorganisms that are attached to a biotic or abiotic surface and that live in self-produced or acquired extracellular polymers (EPS) [8,9]. *L. monocytogenes* is capable of forming biofilms on the surface of food and a variety of materials [10–12]. The presence of biofilms enhances the resistance of these organisms to unfavourable environments, including exposure to UV light, high pH, high temperature and dryness [13]. Furthermore, biofilms exhibit increased resistance to antibiotics, and bacteria within biofilms are difficult to eradicate [14]. The concentration of antibiotics required to eradicate biofilms is typically 100–1000 times the minimum concentration required to kill planktonic cells. In the food industry, biofilms can cause outbreaks of foodborne illness [15,16]. The use of sublethal antibiotic concentrations tends to induce bacterial resistance, necessitating the development of new and effective antibiofilm drugs.

Bacteriocins are defined as ribosomally synthesised peptides produced by bacteria that possess antimicrobial properties against other bacteria [17–19]. Bacteriocins have been the subject of considerable interest as antimicrobial and antibiofilm reagents. Studies have demonstrated that bacteriocins have a significantly inhibit effect on biofilms formed by organisms, including *Pseudomonas aeruginosa*, *Escherichia coli* and *Salmonella enterica* [20,21]. Bacteriocins BacF1 and BacF2 can disrupte biofilms formed by *S. aureus* and *P. aeruginosa* [22]. Bacteriocin BM173 and lactocin AL705 significantly inhibite *L. monocytogenes* biofilm formation [23,24]. Bacteriocin derived from *Lactobacillus plantarum* PTCC 1745 had antibiofilm activity against multidrug-resistant *Acinetobacter baumannii* [25]. Nevertheless, the majority of these studies have concentrated on class I and II bacteriocins, with a notable absence of research on class III bacteriocins and their potential for inhibiting biofilm formation by *L. monocytogenes*.

**Fig 1:**
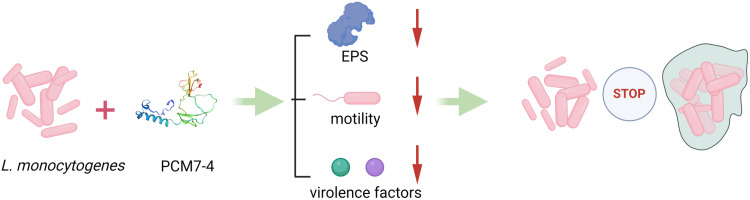
Schematic diagram summarizing the overall mechanism of action.

In our previous study, a novel class III bacteriocin, designated PCM7−4, was isolated and characterised from *Bacillus velezensis* CM7−4, which was isolated from seawater and the minimum inhibitory concentration (MIC) value for bacteriocin PCM7−4 against *L. monocytogenes* was found to be 5.625 μg/mL [26]. In this study we analysed the effects of bacteriocin PCM7−4 on the growth, motility, biofilm production and expression of key biofilm-related genes of *L. monocytogenes*. The overall mechanism of action of this study is shown in Fig 1. The findings of this study might contribute to the development of novel drugs against *L. monocytogenes* biofilms.

## 2. Materials and methods

### 2.1. Bacterial strain and bacteriocin PCM7-4

*L. monocytogenes* (ATCC19115) was cultured in BHI broth and stored at −80 °C with a solution of glycerol at a concentration of 30%. The bacteriocin PCM7−4 was isolated and purified as described previously [26].

### 2.2. Growth curve of *L. monocytogenes* affected by bacteriocin

*L. monocytogenes* was inoculated into BHI broth and incubated overnight at 37 °C. The bacteriocin PCM7–4 was added at concentrations of 1/32 × MIC (0.17578125 μg/mL), 1/16 × MIC (0.3515625 μg/mL), 1/8 × MIC (0.703125 μg/mL), 1/4 × MIC (1.40625 μg/mL), 1/2 × MIC (2.8125 μg/mL), 1 × MIC (5.625 μg/mL), and 2 × MIC (11.25 μg/mL). Samples were collected at 2-hour intervals for a total of 24 hours, and the optical density (OD600 nm) was determined via a microplate reader(KD-870, Kodi Yunxiang Biological Technology).

### 2.3. Quantification of biofilms

The impact of bacteriocin PCM7–4 on biofilm formation was investigated via crystal violet staining. *L. monocytogenes* was inoculated in BHI broth and cultured overnight and diluted to 1 × 10^7^CFU/mL. Bacteriocin was added at concentrations of 1/16 × MIC and 1/8 × MIC, and 200 µL of the culture was added to each well of a 96-well polystyrene microtiter plate (701011, Wuxi NEST Biotechnology Co.,Ltd) and incubated at 37 °C for 24 h to allow biofilm formation. After the supernatant was removed, the wells were washed three times with PBS buffer, dried, and then stained with 200 µL of 1% crystal violet dye for 30 minutes. After the dye was removed and the mixture was washed again with PBS three times, 200 µL of a solution containing 33% acetic acid was added, and the OD595 was determined via a microplate reader (KD-870, Kodi Yunxiang Biological Technology). The control group did not receive bacteriocin treatment.

### 2.4. Analysis of extracellular polymeric substances (EPS)

The impact of bacteriocin PCM7–4 on exopolysaccharides was evaluated through the use of the phenol‒sulfuric acid method as described with minor modifications [27]. Sterile slides (18 mm, borosilicate glass, rotundity) were placed in 24-well plates (702011, Wuxi NEST Biotechnology Co.,Ltd), and *L. monocytogenes* was inoculated in BHI broth at 37 °C overnight. The concentration of PCM7–4 was adjusted to 1/16 × MIC and 1/8 × MIC. One millilitre was subsequently added to the wells of a 24-well plate and cultured at 37 °C for 24 hours. The supernatant was removed and washed with PBS buffer three times before 200 µL of PBS was added; the mixture was shake with an oscillator for 1 minute and subsequently subjected to ultrasonication with ultrasonic frequency of 40KHz for 1 minute. Then 5% phenol, poly-saccharides solution and concentrated sulfuric acid were mixed at a volume ratio of 1:1:5 and incubated in the dark for 20 min. The OD490 value was measured via a microplate reader (KD-870, Kodi Yunxiang Biological Technology).

### 2.5. Scanning electron microscopy (SEM)

The impact of bacteriocin PCM7−4 on the biofilm formation of *L. monocytogenes* was investigated via SEM. A sterile coverslip (9 mm, borosilicate glass, rotundity) was placed in a 48-well plate(748011, Wuxi NEST Biotechnology Co.,Ltd), and 1 mL of *L. monocytogenes* (1 × 10^7^CFU/mL) BHI broth culture containing different concentrations of bacteriocin PCM7−4 (1/16 × MIC, 1/8 × MIC) was added to each well of the plate and incubated at 37 °C for 24 h. After the supernatant was removed, the plate was washed three times with PBS. The biofilms were fixed by incubation with 2.5% glutaraldehyde at 4 °C overnight. Following three additional washes with PBS, the samples were dehydrated via a series of ethanol solutions at various concentrations (30%, 50%, 70%, 85%, and finally 100%), dried with CO_2_, and coated with gold. Finally, the samples were observed via SEM (TESCAN MIRA3 LMU, Czech Republic).

### 2.6. Motility assay

*L. monocytogenes* was cultured overnight at 37 °C in BHI broth. PCM7−4 was incorporated into 20mL of BHI supplemented with 0.5% agar at final concentrations of 0, 1/16 × MIC, and 1/8 × MIC. Once the agar solidified, 1 μL bacterial solution was inoculated at the center of each agar plate. The diameter were measured after being cultured at 37 °C for another 16 hours.

### 2.7. Effect on bacterial viability within biofilms

The impact of bacteriocin PCM7–4 on bacterial activity within biofilms was assessed via a CCK-8 kit (Cell Counting Kit-8, Solarbio life sciences, China). To prepare the biofilm, as previously mentioned, the supernatant was removed after 24 h of culture, followed by three washes with PBS buffer. Subsequently, 100 µL of PBS and 10 µL of CCK-8 reagent were added to each well of the 96-well microtiter plate, and the mixture was incubated at 37 °C for 1 hour. The OD450 was determined using a microplate reader (KD-870, Kodi Yunxiang Biological Technology). The treatment group without bacteriocin served as the positive control.

### 2.8. Effect on bacterial concentration with biofilms

The impact of bacteriocin PCM7−4 on the bacterial count within a biofilm was evaluated. Following a 24-hour incubation period, the supernatant was aspirated, and the samples were washed with PBS buffer three times. Subsequently, 200 µL of PBS was added to each well, and the mixture was agitated for 5 minutes and then subjected to ultrasonic treatment with ultrasonic frequency of 40KHz for an additional 5 minutes. Next, 100 µL of gradient dilution mixture was collected and plated onto BHI solid medium. The plates were then incubated at 37 °C for 16 hours before enumeration.

### 2.9. Quantitative reverse-transcription real-time (RT‒qPCR)

The impact of bacteriocin PCM7−4 on biofilm-related genes was evaluated via RT‒qPCR. *L. monocytogenes* was incubated at 37 °C for 16 hours, after which bacteriocin PCM7−4 was added to achieve concentrations of 1/16 × MIC and 1/8 × MIC. The cultures were subsequently maintained at 180 rpm and 37 °C for 6 hours. The control group was not with bacteriocin. Total RNA was extracted via the RNAiso Plus reagent (TaKaRa, Japan), and cDNA was subsequently synthesised via the Prime Script™ RT reagent Kit (TaKaRa, Japan).The polymerase chain reaction (PCR) was prepared according to the instructions provided with TB Green® Premix Ex Taq II (TaKaRa, Japan), with *16S rRNA* serving as the internal reference gene. Real-time PCR was done with FQD-96X QPCR system (BIOER, Hangzhou Bioer technology co.,ltd). The PCR reactions were conducted in a total volume of 25 μL, comprising 12.5 μL of 2 × TB Green Premix Ex Taq II, 1 μL of 0.4 μM PCR forward primer, 1 μL of 0.4 μM PCR reverse primer, 2 μL of cDNA, and 8.5 μL of distilled water. The real-time PCR conditions were as follows: one cycle at 95 °C for 10 minutes and 40 cycles at 95 °C for 10 seconds, 60 °C for 30 seconds, and 72 °C for 32 seconds. The relative gene expression was determined via the comparative Ct (2-^ΔΔ^Ct) method. The primers used for the tested genes are listed in Table 1. Some Primers was referenced from previous articles and other were designed using Snapgene based on *L. monocytogenes* EGD-e genome (NCBI RefSeq NC_003210.1). The experimental procedure was referenced from previous articles with minor modifications [28].

**Table 1:** List of primers used in this study.

Gene	Function	Primer sequences	Size (bp)
*16S rRNA* [26]	internal reference	F: GATGCATAGCCGACCTGAGA	116
		R: TGCTCCGTCAGACTTTCGTC	
*motA*	flagellar motor protein	F: CAACGCTCGGTGTACTTGG	78
		R: CGCTAAGTTTGTCTGGGTTCG	
*motB* [29]	flagellar motor protein	F: TTCTGTTTGCCTCCAGTTC	197
		R: CTCTTGTTCGTTTGCTTCTTTC	
*plcA* [28]	encode phospholipases	F: TCGGACCATTGTAGTCATCTTG	67
		R: TCACGCAAATTCGGCATGC	
*plcB* [28]	encode phospholipases	F: CGCAGCTCCGCATGATATT	60
		R: TTATCCGCGGACCAACTAAG	
*flaA* [30]	flagellin protein	F: CTGGTATGAGTCGCCTTAG	185
		R: CATTTGCGGTGTTTGGTTTG	
*flgE* [31]	flagellar hook protein	F: CAGCAGGTTCCCCGACTTC	55
		R: CGGCCTTGTAGTGCTGCAT	
*fliG* [32]	flagellar motor switch protein	F: AATCGCGACCGAAGTGGTT	59
		R: CTCGTGCAAGGCGTTGTTT	
*degU* [33]	Transcriptional regulator/quorum sensing	F: GGCGCGTATATTCATCCAC	150
		R: TACCTCGCACTCTCTATGCG	
*argA* [32]	Quorum-sensing response regulator	F: GCAGCCGGACATGAATGG	62
		R: AACCACGCGGATCAAACTTC	
*luxS* [33]	Lux system related to quorum sensing	F: CATTTGATGGCAGAACTTGC	127
		R: TGATTTCGAGTGCATCATCA	
*ltrC*	stress response regulator	F: TACGGCGTCGATGAGATACT	144
		R: GAATGTGTGAACGGCGATAC	
*inlA* [28]	Host cell invasion	F:GAATGTAACAGACACGGTCTCAC	71
		R: TCCCTAATCTATCCGCCTGAAG	
*inlB* [28]	Host cell invasion	F:CGAAAGTACAAGCGGAGACTATC	76
		R: GTTTCTGCAAAAGCATCATCTG	
*hly*	listeriolysin O toxin encoding gene;	F: AGCTCATTTCACATCGTCCA	124
		R: TGGTAAGTTCCGGTCATCAA	

### 2.11. Statistical analysis

All the experiments were carried out with 3 independent replications. Significant differences were analysed by one-way analysis of variance (ANOVA) followed by Tukey’s test for multiple comparisons. The p values (*p < 0.05, **p < 0.01, ***p < 0.001) in the graphs indicate statistically significant differences.

## 3. Results

### 3.1. Effects of PCM7−4 on *L. monocytogenes* planktonic growth

**Fig 2:**
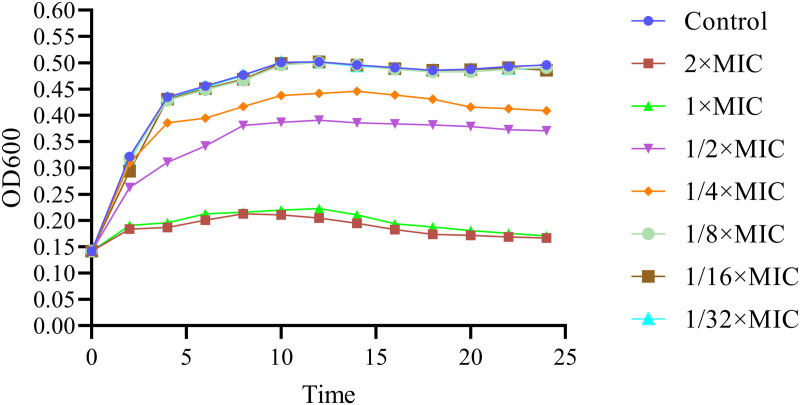
Growth curve of L. monocytogenes exposed to bacteriocin PCM7−4.

The inhibitory effect of bacteriocin PCM7−4 on *L. monocytogenes* was investigated through absorbance growth curve analysis. The findings demonstrated that concentrations of 2 × MIC and 1 × MIC were capable of fully inhibiting bacterial growth, whereas concentrations of 1/8 × MIC, 1/16 × MIC, and 1/32 × MIC had no notable inhibitory effect (Fig 2). Consequently, concentrations of 1/8 × MIC and 1/16 × MIC were selected for further experimentation.

### 3.2. Effects on the biofilm biomass

**Fig 3:**
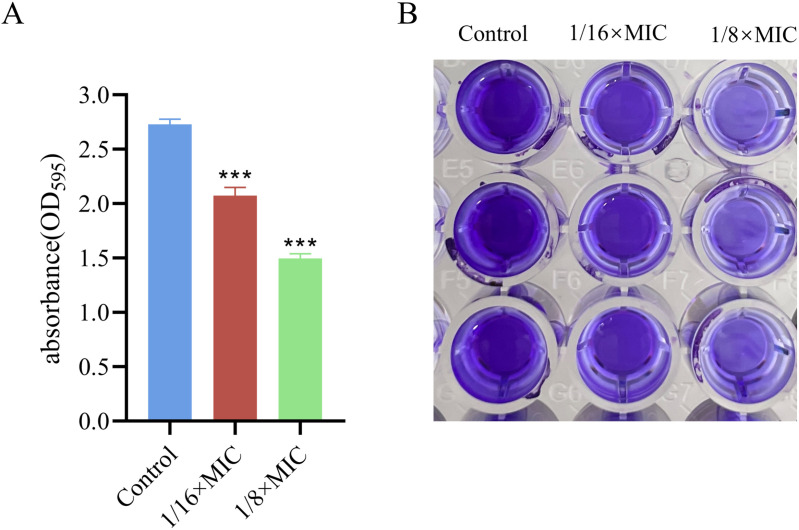
Effects of bacteriocin PCM7−4 on the quantification of biofilm formation in *L. monocytogenes.*

The bacteriocin PCM7−4 exerted a significant and concentration-dependent effect on biofilm biomass. Compared with that in the control group, biofilm formation was reduced by 23.99% ± 1.32% and 45.11% ± 1.53% following treatment with PCM7−4 at concentrations of 1/16 × MIC and 1/8 × MIC, respectively (Fig 3).

### 3.3. Effects on the EPS

**Fig 4:**
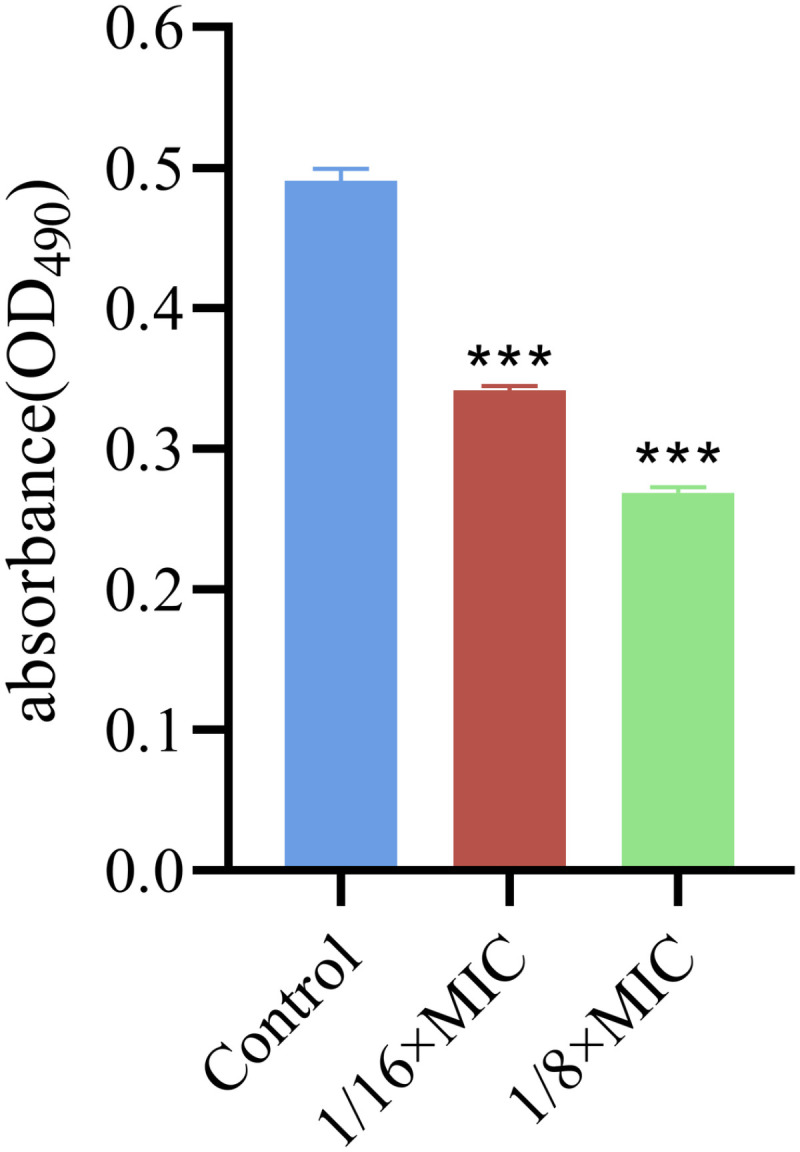
Effects of bacteriocin PCM7−4 on the exopolysaccharide production in *L. monocytogenes.*

Exopolysaccharides constitute essential elements of extracellular polymeric substances (EPS). Compared with those in the control group, the exopolysaccharides were reduced by 30.4% ± 1.65% and 45.27% ± 1.32% following treatment with 1/16 × MIC and 1/8 × MIC concentrations of PCM7−4, respectively (Fig 4).

### 3.4. SEM

**Fig 5:**
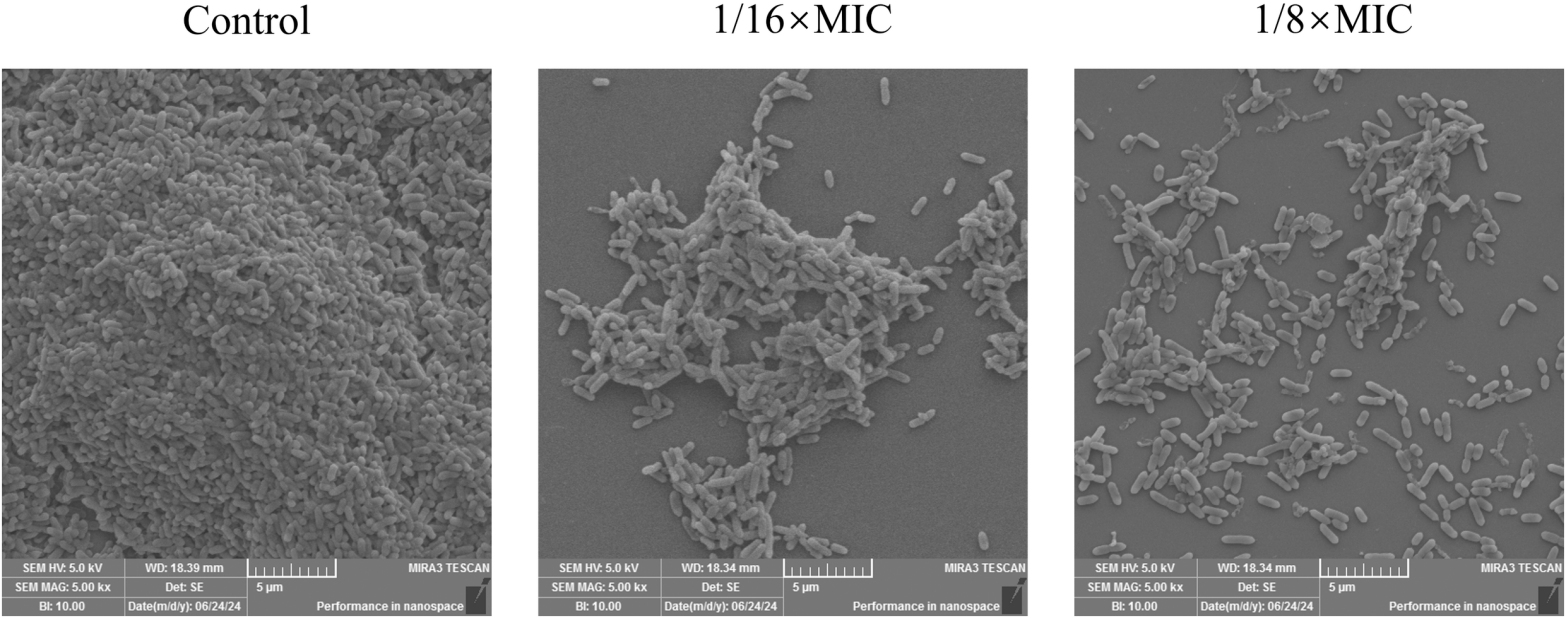
The impact of bacteriocin PCM7−4 on the formation of *L. monocytogenes* biofilms was observed using SEM.

SEM revealed that the cells in the control group exhibited tightly adherent aggregation. The addition of 1/16 × MIC PCM7−4 disrupted biofilm formation, resulting in decreased cell aggregation. Furthermore, the addition of 1/8 × MIC PCM7−4 inhibited biofilm formation and led to dispersed cells (Fig 5).

### 3.5. Effects on motility

**Fig 6:**
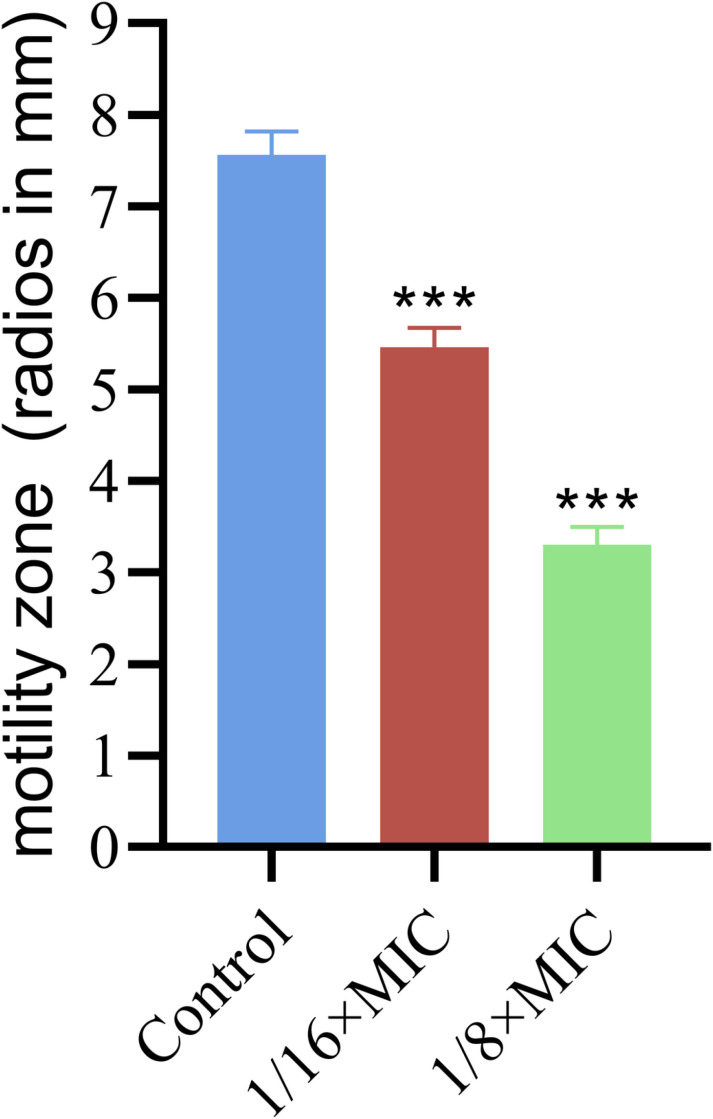
Effects of bacteriocin PCM7−4 on motility in *L. monocytogenes.*

The bacteriocin PCM7−4 was observed to significantly affect the motility of *L. monocytogenes*. Compared with the control group, the treatment group at a concentration of 1/16 × MIC presented a notable reduction in the motility zone of the bacteria, with a mean decrease of 2.1 ± 0.1 mm. Furthermore, the treatment group at a concentration of 1/8 × MIC exhibited an even more pronounced effect, with a mean reduction of 4.27 ± 0.4 mm (Fig 6).

### 3.6. Effects on bacterial viability and concentration within the biofilm

**Fig 7:**
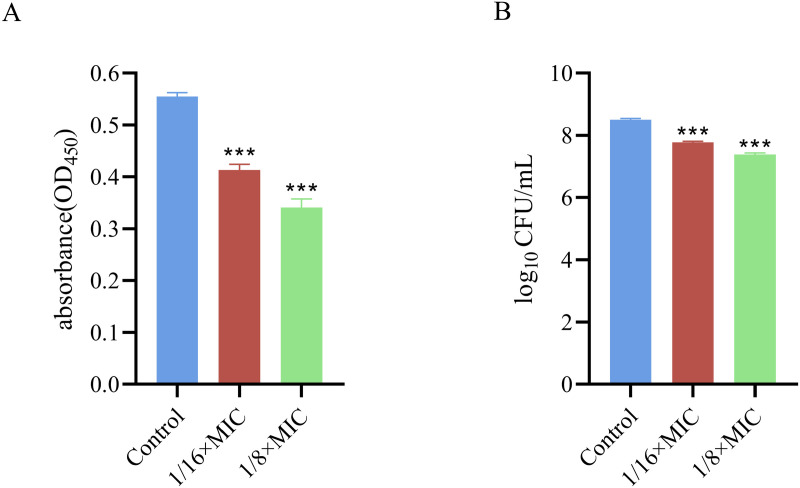
Effects of bacteriocin PCM7−4 on bacteria within the biofilm in *L. monocytogenes.* A: Cell viability B: Bacterial count.

The bacteriocin PCM7−4 had a notable effect on the viability of the cells and the bacterial population within the epidermal envelope of *L. monocytogenes*. The treatment groups with concentrations of 1/16 × MIC and 1/8 × MIC presented 28.6% ± 2.09% and 43.3% ± 2.89% reductions in bacterial viability, respectively (Fig 7A). Additionally, a positive correlation was observed between the bacterial population and viability, with a significant decline in the bacterial population (Fig 7B).

### 3.7. Effects on genes related to biofilm formation

**Fig 8:**
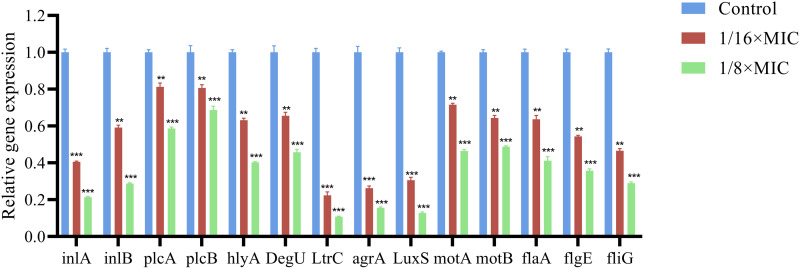
Effects of PCM7−4 on the relative expression of genes associated with biofilm formation.

The bacteriocin PCM7−4 demonstrated efficacy at concentrations of 1/16 × MIC and 1/8 × MIC, resulting in the downregulation of virulence genes associated with *L. monocytogenes* biofilm formation, including *inlA*, *inlB*, *plcA*, *plcB*, and *hlyA*. The expression of *degU*, *ltrC*, population-sensing genes (*agrA*, *LuxS*), and flagellum-associated genes (*flaA*, *flgE*) was found to decrease in a concentration-dependent manner compared with that of the controls (*fliG*, *motA*, *motB*) (Fig 8).

## 4. Discussion

Bacterial biofilms have emerged as significant contributors to global health concerns and are driven by factors such as antibiotic resistance, host immune responses, and external stressors [34]. *L. monocytogenes* and its biofilms represent a significant threat to human health and livestock development. Consequently, the search for novel and effective drugs against *L. monocytogenes* biofilms is of paramount importance. The bacteriocins may be widely used and developed as an anti-biofilm agent in the human health and the development of livestock in the future [35,36]. Many studies have demonstrated the potential of bacteriocins as anti-biofilm agents. Such as, the biofilm inhibition effect of ursoricin on *S. aureus* and *S. epidermidis* [37], plantaricin GZ1–27 can mitigate methicillin-resistant *S. aureus* (MRSA) biofilm formation [38], LAB bacteriocins can combating bacterial biofilms in food processing environments [39].

Sub-inhibitory concentration (SIC) was defined as concentrations not inhibiting bacterial growth [28]. To demonstrate that the inhibition of biofilm formation by PCM7−4 does not involve direct bacterial killing or inhibition of bacterial growth., and gain a more precise understanding of the effects of bacteriocin PCM7−4 on the biofilm, 1/16 × MIC and 1/8 × MIC were selected for subsequent experiments. The results of crystal violet staining demonstrated that the 1/16 × MIC and 1/8 × MIC of bacteriocin PCM7−4 had a pronounced inhibitory effect on biofilm formation. In other studies, demonstrated the inhibitory activity of SICs of bacteriocin BM173、BMP32r and four plant-derived antimicrobials on the biofilm formation of *L. monocytogenes* [23,28,31]. Previous research has also indicated that nisin exerts a considerable inhibitory effect on the biofilm formation of *S. aureus* and *P. aeruginosa* through crystal violet staining [40]. *L. plantarum* subsp. *argentoratensis* SJ33 was observed to produce bacteriocins with notable inhibitory effects on the biofilm of *P. aeruginosa* and *S. aureus* [41]. The biofilm is primarily composed of EPS and bacteria, with the EPS comprising extracellular DNA (eDNA), proteins, and extracellular polysaccharides [42]. Extracellular polysaccharides play a pivotal role in maintaining the intricate spatial configuration of biofilms, impeding the permeation of antimicrobial agents into biofilms and increasing biofilm resistance to drugs [43,44]. The impact of bacteriocin PCM7−4 on the development of the biofilm can be evaluated by determining the concentration of extracellular polysaccharides. The findings of the present study indicate that bacteriocin PCM7−4 at concentrations of 1/16 × MIC and 1/8 × MIC has a notable inhibitory effect on extracellular polysaccharides. A study reported that a bacteriocin-like substance isolated from *L. plantarum* Z102-E inhibited extracellular polysaccharides by 75.55% and 67.51% at concentrations of 3.2 mg/mL and 1.6 mg/mL, respectively [45]. In another study, chitosan was demonstrated to reduce the secretion of extracellular polysaccharides by foodborne *Vibrio parahaemolyticus* [46]. Furthermore, the impact of bacteriocins on EPS can be more readily identified through the use of SEM. In this study, the bacteriocin PCM7−4 was observed to exert a pronounced inhibitory effect on EPS, with a discernible concentration-dependent response. Bacterial motility is intimately linked to the development of biofilms, and disassociating bacterial motility from the flagellar structure of bacteria is challenging [47–49]. Motility is linked to cell surface attachment and subsequent biofilm formation during the initial adhesion stage, and it contributes to resistance to antibacterial substances and the host immune system [50]. Good motor ability and chemotaxis are beneficial for bacteria to obtain more nutrients from the environment or the host and beneficial for bacteria to form biofilms [51]. Flagellar motility is critical for *L. monocytogenes* biofilm formation [52]. In this study, we investigated the impact of bacteriocin PCM7−4 on bacterial motility through a swimming experiment. The results demonstrated that PCM7−4 significantly influenced the motility of *L. monocytogenes*. To further elucidate the underlying mechanisms, we examined the effects of PCM7−4 on bacterial flagellum-related genes through RT‒qPCR. The results indicated that PCM7−4 markedly reduced the expression of flagellum-related genes (*flaA*, *flgE*, *fliG*, *motA*, *motB*). By controlling flagella expression, bacteriocin PCM7−4 can interfere with bacterial movement and preliminary attachment, thereby inhibiting biofilm formation in one study, eugenol was observed to reduce biofilm formation by decreasing bacterial motility, thereby reducing biofilm formation [53]. In other study, Paenibacterin can inhibited bacteria movement by down-regulating flagellar expression, thus inhibiting *L. monocytogenes* biofilm formation [54]. In addition to its impact on EPS, the effect on bacteria within the biofilm is a significant factor in inhibiting biofilm formation. In the present study, bacteriocin PCM7−4 had a concentration-dependent effect on both bacterial viability and biofilm number. The formation of biofilms by *L. monocytogenes* is a complex process that is influenced by a range of factors, including environmental conditions, virulence factors, quorum sensing (QS) and other regulatory mechanisms [30]. Group sensing and virulence factors are highly important in biofilm formation. Group sensing has a considerable influence on the formation and cleavage of biofilms, whereas certain virulence factors play a nonnegligible role in bacterial adhesion and biofilm development [28,32,55]. In this study, bacteriocin PCM7−4 significantly reduced the expression of group-sensing genes (*agrA* and *luxS*) and virulence factors (*inlA*, *inlB*, *plcA*, *plcB*, and *hly*). Similar inhibitory effects of the melittin peptide and bacteriocin BMP32r on the expression of these genes have been previously reported [28,30]. The bacteriocin PCM7−4 had a significant inhibitory effect on *L. monocytogenes* biofilm formation, and PCM7−4 was promising to be developed as an antibiofilm agent against *L. monocytogenes*. However, the current process and efficiency of isolating and obtaining the bacteriocin PCM7−4 are insufficient, and the precise mechanism by which PCM7−4 inhibits *L. monocytogenes* biofilm formation remains to be elucidated.

## 5. Conclusion

The present study demonstrated that bacteriocin PCM7−4 was effective in inhibiting and killing the planktonic bacteria of *L. monocytogenes*. Bacteriocin PCM7−4 was observed to significantly inhibit biofilm formation, which was achieved by decreasing extracellular polysaccharide production, inhibiting bacterial motility, and decreasing flagellar, community sensing, and virulence factor gene expression. Consequently, PCM7−4 could be developed as an anti-*L. monocytogenes* biofilm drug. However, further studies on the mechanism of action of PCM7−4 against biofilms are needed.
